# Theta Synchrony Is Increased near Neural Populations That Are Active When Initiating Instructed Movement

**DOI:** 10.1523/ENEURO.0252-20.2020

**Published:** 2021-01-06

**Authors:** Ashwin G. Ramayya, Andrew I. Yang, Vivek P. Buch, John F. Burke, Andrew G. Richardson, Cameron Brandon, Joel M. Stein, Kathryn A. Davis, H. Isaac Chen, Alexander Proekt, Max B. Kelz, Brian Litt, Joshua I. Gold, Timothy H. Lucas

**Affiliations:** 1 Department of Neurosurgery, Perelman School of Medicine, University of Pennsylvania, Philadelphia, PA 19104; 2 Department of Neurosurgery, University of San Francisco, San Francisco, CA 94143; 3 Department of Neuroscience, Perelman School of Medicine, University of Pennsylvania, Philadelphia, PA 19104; 4 Department of Radiology, Perelman School of Medicine, University of Pennsylvania, Philadelphia, PA 19104; 5 Department of Neurology, Perelman School of Medicine, University of Pennsylvania, Philadelphia, PA 19104; 6 Department of Anesthesiology, Perelman School of Medicine, University of Pennsylvania, Philadelphia, PA 19104

**Keywords:** high frequency activity, instructed movement, intracranial EEG, sensory-motor transformation, synchrony, theta

## Abstract

Theta oscillations (3–8 Hz) in the human brain have been linked to perception, cognitive control, and spatial memory, but their relation to the motor system is less clear. We tested the hypothesis that theta oscillations coordinate distributed behaviorally relevant neural representations during movement using intracranial electroencephalography (iEEG) recordings from nine patients (*n* = 490 electrodes) as they performed a simple instructed movement task. Using high frequency activity (HFA; 70–200 Hz) as a marker of local spiking activity, we identified electrodes that were positioned near neural populations that showed increased activity during instruction and movement. We found that theta synchrony was widespread throughout the brain but was increased near regions that showed movement-related increases in neural activity. These results support the view that theta oscillations represent a general property of brain activity that may also play a specific role in coordinating widespread neural activity when initiating voluntary movement.

## Significance Statement

Whereas theta oscillations in the human brain have been extensively related to a wide range of perceptual and cognitive functions, there is only limited data linking theta oscillations to motor systems. In this study, we use intracranial electroencephalography (iEEG) to show that theta oscillations (3–8 Hz) are widespread throughout the brain but further increased near movement-related neural populations during instructed movement. Our results provide a link between theta oscillations and motor systems.

## Introduction

Theta oscillations (3–8 Hz) in the human brain have been linked to a wide range of perceptual and cognitive functions, but their relation to the motor system is less clear (Buzsáki, 2006; Cavanagh and Frank, 2014; Jacobs, 2014; VanRullen, 2016). It has been hypothesized that theta oscillations provide a mechanism for temporally coordinating widespread sensory, goal-related, and motor neural populations that are behaviorally relevant for voluntary movement (Cavanagh et al., 2012).

In support of this view, a recent scalp electroencephalography (EEG) study observed that theta oscillations were phase locked to the initiation of voluntary movement and also related to performance on a visual discrimination task (Tomassini et al., 2017). These data suggest that theta oscillations are related to both sensory and motor behaviors, possibly playing a specific role in coordinating widespread sensory and motor neural activity during voluntary movement. However, because of the limited spatial resolution of scalp EEG, these data do not speak to the anatomic relationship between theta oscillations and brain regions containing neural populations that are behaviorally relevant for movement (e.g., sensory, goal-related, and motor). As an alternative explanation, it is possible that theta oscillations are anatomically widespread and independent from these behaviorally relevant neural populations, suggesting an indirect relationship to voluntary movement, rather than a direct role in coordinating movement-related neural populations

We studied whether theta oscillations specifically synchronize regions that are behaviorally relevant for initiating voluntary movement by obtaining intracranial EEG (iEEG) recorded from patients with drug-refractory epilepsy as they performed a simple instructed movement task (Parvizi and Kastner, 2018). high frequency activity (HFA; 70–200 Hz, often labeled “high gamma”) from iEEG data provides a measure of local firing activity with high spatial and temporal resolution (Manning et al., 2009; Ray and Maunsell, 2011; Burke et al., 2015; Dubey and Ray, 2019) and can be used to identify electrodes near sensory and motor neural populations (Cogan et al., 2014; Flinker et al., 2015). Moreover, low frequency components of iEEG data, such as theta, can measure widespread network changes that can also be observed at the level of scalp EEG (Buzsáki, 2006; Burke et al., 2013; Solomon et al., 2017; Bickel et al., 2018). Our approach was to use task-related HFA increases to identify electrodes that were in proximity to task-related neural populations and then assess interactions between these regions by measuring theta synchrony between them (Solomon et al., 2017, 2019)

We grouped electrodes based on their proximity to distributed behaviorally relevant neural populations based on patterns of cue-evoked HFA. These included a widely distributed “instruction-related” group of electrodes that showed HFA increases during instruction presentation and a perirolandic-localized “movement-related” group of electrodes that only showed HFA increases during movement, suggesting proximity to sensory/goal-related neural populations and movement-related neural populations, respectively. We found that theta synchrony occurred between widespread brain regions, including regions that did not show HFA increases, consistent with a general role in brain function. However, theta synchrony was further increased near regions that showed movement-related HFA increases. These results support the view that theta oscillations play a role in coordinating distributed neural activity when initiating voluntary movement.

## Materials and Methods

### Subjects

Patients with drug-refractory epilepsy underwent a surgical procedure in which grid, strip, and depth electrodes were implanted to localize epileptogenic regions (Table 1). Clinical circumstances alone determined the number and placement of implanted electrodes. Data were collected from our institution and was approved by the Institutional Review Board. Informed consent was obtained from all participants. In total, we recorded neural activity from nine subjects (ages 21–53; three females, six males). None of these patients had seizures originating from sensory or motor cortex. We did not specifically exclude electrodes based on epileptic activity (see Discussion, Limitations)

**Table 1:** Subject table

Subject #	Age of time of implant	Gender	Number of electrodes	Number of task-responsive	Number of electrodes with theta oscillations
1	53	Male	45	31	10
2	24	Male	81	61	45
3	35	Female	31	20	2
4	28	Male	87	22	19
5	30	Male	48	19	9
6	24	Male	64	42	5
7	29	Male	79	66	35
8	40	Female	11	8	2
9	21	Female	44	19	7

### Instructed movement task

**Figure 1.**
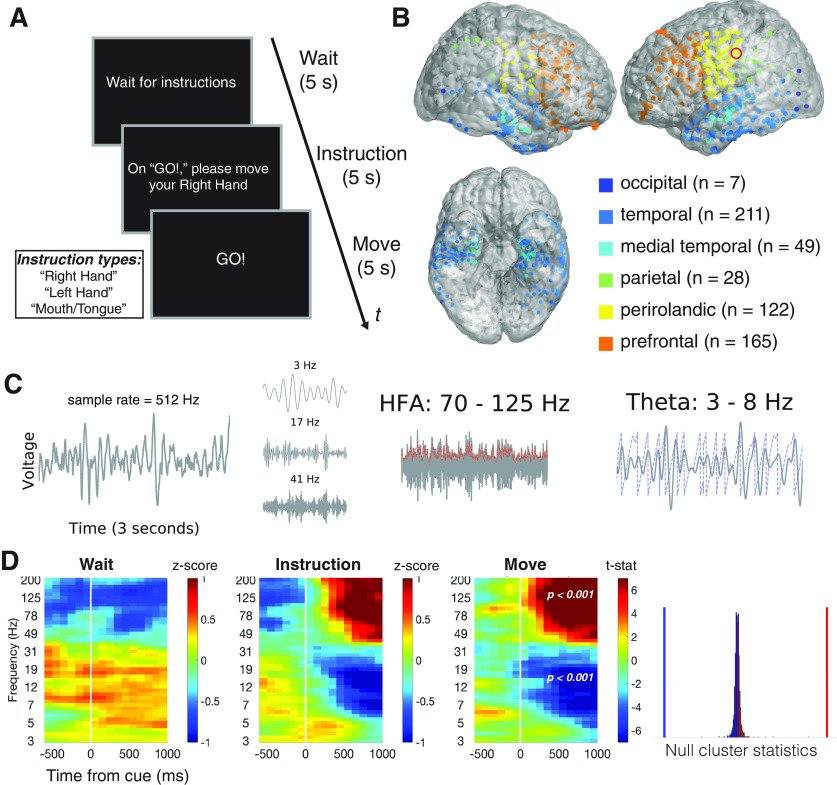
Methods. ***A***, Instructed movement task. Each trial consisted of three cues: a “wait” cue, an “instruction” cue (where subjects were instructed to prepare of three movements: move their right hand, left hand or mouth and tongue), and a “go” cue movement (referred to as “movement-cue” throughout the text), where subjects were asked to carry out the instructed movement. ***B***, We recorded from 490 bipolar electrode pairs (henceforth, “electrodes”) throughout neocortex and medial temporal lobe that are shown here in standard MNI152 space (henceforth, “brain plot”). ***C***, We illustrate our methods of extracting neural activity using a three second voltage recording from an example electrode in perirolandic cortex (indicated by a red circle in panel ***B***). We used wavelet convolution of voltage time series to extract time-resolved measures of power and phase at a wide range of frequencies (3–200 Hz). We illustrate three example wavelet convolutions (3, 17, 41 Hz). We focus our analyses on two neural signals: (1) HFA, power in wideband 70- to 125-Hz frequencies, which is an established correlate of aperiodic local spiking activity (dashed red line indicates power estimates); and (2) theta oscillations, periodic membrane potential fluctuations in narrowband 3 to 8 Hz frequencies, that are hypothesized to facilitate interregional neuronal interactions (dashed blue line indicates phase estimates). ***D***, We identified task-responsive electrodes using a non-parametric cluster-based statistical procedure that identified significant task-related power changes in any contiguous time-frequency interval. We illustrate our method to detect movement-related changes in power (“move”–“wait”) at an example electrode (same as ***C***). Time-frequency plots summarizing data across various time and frequency windows (3–200 Hz; centered from −500 to 1000 ms relative to cue onset). We show average cue-locked power (left, “wait”; center-left, “move”) or cue-locked power differences (center-right, *t* statistics comparing “move” and “wait” conditions). Power values have been log transformed and normalized within frequency (“whitened”). White line indicates cue onset. We obtained non-parametric *p* values for each time-frequency cluster by comparing effect size to a null distribution of cluster statistics (right). Vertical blue line marks effect size of low frequency power decrease relative to null distribution, whereas vertical red line marks high frequency power increase. We considered an electrode to be task-responsive if it showed a significant instruction or movement-related power change in any time-frequency range.

Subjects were asked to perform a simple instructed motor task at the bedside during the epilepsy monitoring period, ranging from 2 to 14 days after implantation. In each trial, subjects were asked to move either their right hand, left hand, or mouth and tongue (henceforth, “specific movements”). They were presented with written instructions on a laptop screen. Before the task, hand movements were demonstrated as opening and closing the hand and mouth and tongue movements were demonstrated as repetitive movement of the jaw and tongue without producing words or sound. Each trial consisted of three cues presented in sequence: a wait cue (“wait for instructions”), an instruct cue (e.g., “on GO! please move your right hand”), and a move cue (“GO!”; Fig. 1*A*) Each screen was displayed for 5 s with 24 trials per session. We could not perform behavioral analyses as reaction times were not recorded during the task.

### Electrocorticographic recordings

We recorded iEEG from Ad-tech subdural (grids and strips, 4-mm contacts, spaced 10 mm apart) and intraparenchymal depth electrode (1.1-mm diameter, four contacts spaced 5 mm apart). Intraparenchymal depth electrodes were typically placed in medial temporal lobe structures but also used to target frontal lobe structures (subjects 2, 4, and 11). iEEG was recorded using a Nicolet or Natus EEG system. Based on the amplifier and the discretion of the clinical team, signals were sampled at either 250 or 512 Hz. Signals were converted to a bipolar montage by taking the difference of signals between each pair of immediately adjacent electrodes on grid, strip, or depth electrodes. The resulting bipolar signals were treated as new virtual electrodes (henceforth, “electrodes”), originating from the midpoint between each electrode pair (Burke et al., 2013). Analog pulses synchronized the electrophysiological recordings with stimulus presentation events. We excluded electrodes that recorded prominent 60-Hz electrical line noise, defined as electrodes that showed greater spectral power in 58- to 62-Hz range as compared with the 18- to 22-Hz range. Subject 5 underwent a montage change in between sessions resulting in different electrode labels. We only included data from the original montage.

### Anatomical localization

Intracranial electrodes were manually identified on each postoperative CT scans. To map electrode coordinates from the CT scan onto the cortical surface, we registered each postoperative CT scan to each patient’s preoperative MRI scan using a rigid-body 6 degrees of freedom affine transformation algorithm, and manually adjusted each transform such that electrodes were positioned as close to the cortical volume as possible. We co-registered each patient’s preoperative MRI scan to the MNI152 brain to obtain anatomic labels (Jenkinson et al., 2012). Based on MNI152 labels, electrodes were manually assigned to one of several regions: prefrontal, perirolandic, parietal, temporal, medial temporal, or occipital (Fig. 1*B*). Electrodes that remained unlabeled based on the co-registration to the MNI152 volume were manually assigned to one of these locations.

### Extracting spectral power

We extracted 3 s segments of iEEG data from 1000 ms before and 2000 ms after each cue presentation (“wait” cue, the “instruction” cue, and the “Go” cue). We extracted spectral power with 50 complex valued Morlet wavelets (wave number 7) with center frequencies logarithmically spaced from 2 to 200 Hz (Addison, 2002). We first squared and then log-transformed the wavelet convolutions, resulting in a continuous representation of log-power surrounding each cue presentation. We averaged these log-power traces in 500-ms epochs with 400-ms overlap surrounding the presentation of each task related cue (Fig. 1*C*). We z-transformed power at each frequency by the mean and standard deviation of power values obtained from randomly selected clips of iEEG data recorded from that session so as to not bias values toward any particular task-related event (Burke et al., 2013; Ramayya et al., 2015).

### Identifying electrodes that showed task-responsive activity

We identified “task-responsive” electrodes as those that showed significant spectral power changes at any frequency in relation to the instruction or movement cue. For each electrode, we identified spectrally and temporally contiguous power differences between task conditions by performing a cluster-based permutation procedure that accounts for multiple comparisons (Maris and Oostenveld, 2007). We describe the statistical procedure for the comparison between movement and wait intervals (“move–wait”), but separately performed this procedure comparing instruction and wait intervals (“instruct–wait”). As suggested by Maris and Oostenveld, we began by performing an unpaired *t* test at each time interval comparing power distributions associated with all movement and wait trials performed by the subject. Using an uncorrected *p *=* *0.05 as a threshold, we identified the largest cluster of adjacent time-frequency windows that showed positive *t* statistics (greater power following movement compared with wait trials), and the largest cluster of adjacent time-frequency windows that showed negative *t* statistics (greater power during wait trials compared with move trials). By taking the sum within each of these clusters, we computed positive and negative “cluster statistics,” respectively. To assess the statistical significance of each cluster statistic, we generated a null distribution of cluster statistics based on 1000 iterations of shuffled data (on each iteration, “move” and “wait” labels were randomly assigned to power values recorded during the session). Based on where each cluster statistic fell relative to the null distribution, we generated a one-tailed *p* value for each effect, that we converted to a two-tailed *p* value. For instance, a clustered power increase with a one-tailed *p* value of 0.025 was assigned a two-tailed *p* value of 0.05, corresponding to a 5% false-positive rate of identifying either a positive or negative cluster at that electrode. We considered an electrode to be task-responsive if we observed a cluster statistic with a *p *<* *0.05 during either the move–wait comparison or the instruct–wait comparison (Fig. 1*D*).

### Grouping electrodes based on cue-evoked HFA

We grouped task-responsive electrodes based on HFA changes in relation to the instruction cue and the movement cue. For each electrode, we measured HFA as average *z*-scored, log-transformed power of wavelets ranging from 70 to 125 Hz (log transform before *z*-score, see above, Extracting spectral power). We defined a baseline interval as the 500 ms before the wait cue. We performed a paired *t* test between mean HFA values during the 0- to 1000-ms time interval after the instruction cue and this prewait baseline interval to measure instruction-evoked HFA. Similarly, we performed a paired *t* test between mean HFA values during the 0- to 1000-ms time interval after the movement cue and this prewait baseline interval to measure movement-evoked HFA. We grouped electrodes based on evoked HFA changes as follows. First, we identified instruction-related electrodes as those that showed increased HFA following the instruction cue (*t *>* *2.5, *p *<* *0.05). Second, we identified movement-related electrodes as those that showed increased HFA following the movement cue (*t *>* *2.5, *p *<* *0.05), but that did not show instruction-related HFA increases. Third, we identified “HFA decrease” electrodes as those that showed decreased HFA either during the instruction cue or the movement cue (*t* < −2.5, *p *<* *0.05). Finally, we labeled all remaining task-responsive electrodes as “HFA null” as they did not show significant cue-related changes in HFA during the task.

We selected these grouping criteria as a method to generally distinguish distinct patterns of local neural activity observed in this dataset, with the acknowledgment that specific boundary criteria are arbitrary. We used this data-driven approach to identify electrode groups rather than a region of interest analysis because some patterns of local neural activity might be widely distributed throughout the brain, and because a particular region might contain distinct and opposing patterns of neural activity (Ramayya et al., 2015). We grouped electrodes that showed both instruction-related and movement-related increases in HFA as part of the instruction-related group, rather than the movement-related group. This response pattern likely reflects a combination of visually responsive neural populations (that show increases in activity following both cues) but may also include preparatory motor populations that show increased activity during movement instruction and execution.

### Identifying theta oscillations at each electrode

We identified oscillations at each electrode by assessing whether the power spectrum showed narrowband peaks above the 1/*f* background activity using a recently described parametric curve-fitting method (Donoghue et al., 2020). This approach has two main advantages over the simpler alternative of averaging power in narrowband frequency ranges. First, it avoids conflating any potential oscillatory components with the aperiodic background component of the power spectrum, which has been shown to reflect asynchronous neural spiking and noise (Manning et al., 2009; Ray and Maunsell, 2011; Voytek and Knight, 2015; Dubey and Ray, 2019). Second, this approach does not require predefined frequency ranges to identify oscillatory spectral peaks in the power spectrum and can account for electrode-to-electrode variability in the center frequency of oscillations.

To apply this method, we concatenated iEEG data from each trial during the entire time interval (0–5000 ms following the wait, instruction, and move cues) into a single time series for the entire recording. We computed the power spectrum of this time series using Welch’s method for frequencies ranging from 2 to 50 Hz. Briefly, we fit the aperiodic 1/*f* background component of the power spectrum using an exponential function (in log power vs linear frequency space) and then fit Gaussian peaks to the residual “flattened” power spectrum to assess for oscillatory peaks [python fitting oscillations and one over f (*FOOOF*) package Donoghue et al., 2020]. We labeled electrodes as recording theta oscillations if we observed a spectral peak with a center frequency in the 3 to 8 Hz range.

### Measuring pairwise theta phase synchrony between electrodes

We studied pairwise connectivity between electrode pairs by measuring theta phase synchrony using methods similar to a recent study of medial temporal lobe theta (Solomon et al., 2019).

We used wavelets to extract instantaneous theta phase for each electrode throughout the task. We convolved the iEEG signal from each electrode with complex-valued Morlet wavelets from 3–8 Hz (wave number = 5, 6 wavelets spaced 1 Hz apart). Each wavelet was convolved with 6000 ms of data surrounding the instruction and movement cue (−1000 to 5000 ms surrounding each cue) and buffered with 1000 ms at the beginning and end of each segment (clipped after convolution). We averaged phase values across wavelets (circular mean) resulting in a single theta phase value for each time sample at each electrode.

For a given pair of electrodes, we measured within-trial theta phase coupling across time in 1000-ms time intervals spanning the trial epoch. This duration allows for at least three cycles of a theta oscillation. We focused on two time intervals that we hypothesized would be important for coordinating neural populations for voluntary movement: the 1000 ms surrounding the instruction cue and the move cue. We centered each of these time intervals from 250 ms before the cue to 750 ms following the cue to account for any temporal smearing into the prestimulus interval from the wavelet convolution. We also present data from surrounding time intervals using a sliding window analysis (ranging from −1000 ms to cue, to 1000 to 2000 ms postcue).

For a given time interval, we computed theta phase differences between the electrodes for each sample of time. The null hypothesis states that the distribution of phase differences across trials between two unrelated signals should be uniformly distributed on a unit circle. We assessed the non-uniformity (“tightness”) of the distribution of phase differences for each pair at each time interval by computing the resultant vector length (RVL; python *circstat* package, Circular Statistics Toolbox; Berens and Velasco, 2009). RVL values can range from 0 (uniform circular distribution suggesting independent phases) to 1 (non-uniform distribution suggesting high phase coupling). We obtained a RVL value separately for each trial for the given time interval that we refer to as the “true” RVL distribution in the section below.

We used a non-parametric resampling procedure to estimate the null distribution of RVL values for a given pair of electrodes as follows. For each iteration, we randomly selected a 1000 ms clip of phase values from each electrode that had an intact autocorrelation structure but were mismatched in trial number and in temporal relation to cue presentation. This method ensured that the null distribution would not be influenced by event-related phase reset phenomena occurring at both electrodes. First, we randomly select a trial for each electrode, then circularly shifted each 6000-ms phase clip by a random value, and then selected a contiguous 1000 ms clip for each electrode. We computed a null RVL value by comparing phase differences between these random phase clips. We repeated this procedure 1000 times resulting in a null RVL distribution for a given electrode pair. To measure the extent to which phase coupling was greater than expected by chance during a given time interval, we performed an unpaired *t* test between true RVL distribution and the null RVL distribution. We refer to the resulting *t* statistics as “synchrony *t* statistics” when presenting results, and specifically refer to “instruction synchrony *t* statistics” and “movement synchrony *t* statistics” for the instruction-related and movement-related time intervals, respectively. We computed a *t* statistic to assess whether the distribution of RVL values across trials was greater than expected by chance, rather than the mean RVL value that might be heavily influenced by a handful of outlier trials.

### Statistical tests

We performed ANOVA or Student’s *t* tests to compare continuous distributions and χ^2^ test to compare categorical distributions. We performed false discovery rate (FDR) correction for multiple comparisons (Benjamini and Hochberg, 1995). We considered an FDR-corrected *p* value < 0.05 to be statistically significant. We also occasionally report uncorrected *p* values as noted. We performed analyses using MATLAB and Python using both publicly available packages (e.g., *NumPy*, for numerical computing; *SciPy*, for statistics and signal processing; *MNE*, for spectral analyses; *pycircstat*, for circular statistics; *FOOOF*, fitting oscillations and one over f, for power spectrum modeling; and *statsmodels*, for regression modeling) and custom code.

To assess whether task-related neural activity differed in relation to specific movements (left hand vs right hand vs mouth and tongue), we applied a one-way ANOVA on the distribution of mean power within the time-frequency range of the clustered power change at each electrode (uncorrected *p *<* *0.01).

### Data sharing

Behavioral and neural data obtained for this study and associated code will be made available on request

### Code accessibility

The code described in this paper is freely available online at https://github.com/ashwinramayya/code_RamaEtal20_sensorymotor.

## Results

### Identifying task-related electrodes

We obtained iEEG recordings from nine patients as they performed a simple instructed movement task with distinct instruction and movement intervals (Fig. 1*A*). We recorded from 490 bipolar electrode pairs across widespread brain regions (Fig. 1*B*). We excluded 70 electrodes that showed prominent electrical line noise.

We found that 288 of the remaining electrodes were task-responsive, in that they showed a spectral power change in relation to the instruction or movement cue, which was more frequent than expected by chance (χ^2^ statistic = 336, *p *<* *0.001; 23.4 expected based on the 5% false-positive rate). We identified these power changes without specifying a time interval or frequency range using a non-parametric statistical procedure cluster-based procedure (Maris and Oostenveld, 2007). Briefly, this method assessed whether an electrode showed consistent cue-related power changes in any contiguous time-frequency window that were greater in magnitude than expected by chance. We illustrate this method in Figure 1*B–D* using an example electrode from the perirolandic cortex that showed an increase in HFA and a decrease in wideband low-frequency power during the movement interval as compared with the wait interval (FDR-corrected *p*’s < 0.001)

### HFA identified distinct neural response functions throughout the brain

**Figure 2.**
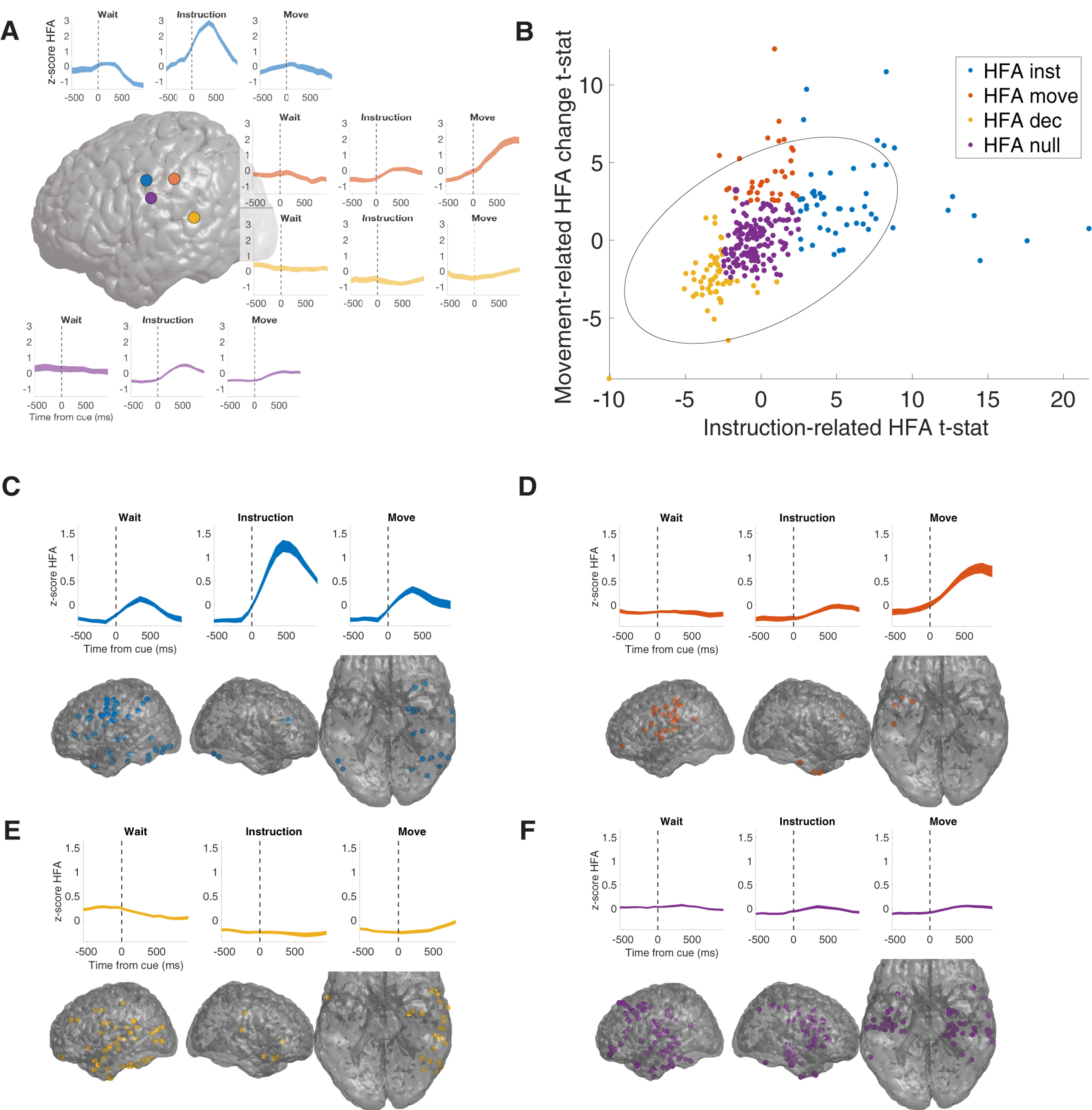
HFA identifies distinct local neural responses. ***A***, Four example electrodes showing distinct HFA responses during the task from the same subject. Brain plot illustrates the location of each electrode (purple, blue, and orange are in perirolandic, whereas yellow is parietal, on the border between postcentral gyrus and superior temporal sulcus). The HFA responses in each of these electrodes correspond to the average response functions described below. ***B***, Distribution of instruction and movement-related HFA across all electrodes (see main text for details). We identified four groups of electrodes as follows: (1) Instruction-related electrodes (blue, 53 electrodes, 7 subjects), (2) Movement-related electrodes (red, 34 electrodes, 6 subjects), (3) HFA decrease (yellow, 54 electrodes, 8 subjects), and (4) HFA null (purple, 147 electrodes, 9 subjects). Gray error ellipse marks 95% confidence interval of bivariate distribution. ***C–F***, Average HFA (*z*-scores) at each electrode group and their anatomic distributions on brain plots.

We identified electrodes that were positioned near behaviorally relevant neural activity using task-related HFA changes (70- to 125-Hz power), a known proxy for local neural firing rates (Dubey and Ray, 2019). At each task-responsive electrode, we studied how HFA changed following the instruction and move cues to estimate the response function of nearby neural populations. We illustrate the high spatial and temporal specificity of HFA by showing distinct response functions from four nearby electrodes from an example subject (Fig. 2*A*). Two electrodes showed large time-locked HFA increases (blue electrode only during instruction and orange electrode only during movement). The other two electrodes did not show large HFA changes (yellow showed a small magnitude decrease following instruction and movement, whereas purple showed no reliable change).

We grouped electrodes based on cue-related HFA changes to generally distinguish distinct patterns of neural activity observed in this dataset (Fig. 2*C–F*). We quantified the HFA changes at each electrode by using *t* tests to compare mean HFA during the 1000 ms following each cue to a baseline interval (500 ms preceding the wait cue), resulting in an instruction-related *t* statistic and a movement-related *t* statistic. We grouped electrodes into one of four mutually exclusive groups as follows (HFA electrode groups). First, instruction-related electrodes were those that showed increased HFA following the instruction cue (*n* = 53 electrodes, 7 subjects) (Fig. 2*C*). Second, movement-related electrodes were those that showed only increased HFA following the movement cue (*n* = 34 electrodes, 6 subjects) (Fig. 2*D*). Third, HFA decrease electrodes were those that showed decreased HFA either following either cue (*n* = 54 electrodes, 8 subjects) (Fig. 2*E*). Fourth, HFA null electrodes were task-responsive electrodes that did not show reliable cue-related HFA changes (*n* = 147 electrodes, 9 subjects) (Fig. 2*F*).

**Figure 3.**
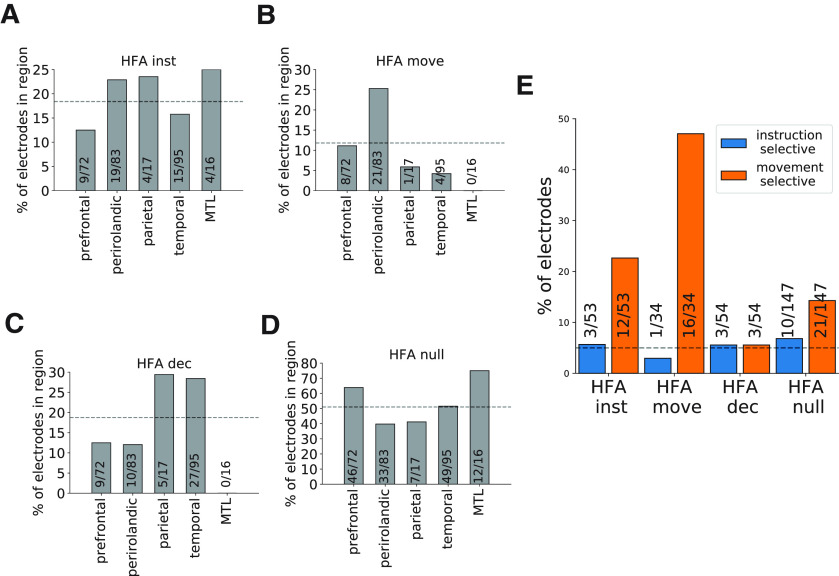
HFA electrode groups have distinct anatomic and functional properties. ***A–D***, Anatomical distribution of each group across five regions of interest (prefrontal, perirolandic, parietal, temporal, and MTL). ***E***, Rates of instruction and movement selectivity across each electrode group. Dashed horizontal lines indicate the overall frequency of observing an electrode from the specified group in any brain region (***A–D***), and the false positive rate of observing selective electrodes (***E***).

HFA electrode groups showed distinct anatomic distributions (Fig. 3). Movement-related electrodes (Fig. 3*B*) were heavily clustered in perirolandic regions (*n* = 21/34, χ^2^ statistic = 19.8, *p *<* *0.001), whereas instruction-related (Fig. 3*A*) and HFA null electrodes (Fig. 3*D*) were widely distributed throughout the brain (χ^2^ test, *p*s *>* 0.15). HFA decrease electrodes (Fig. 3*C*) were also widespread but were more frequently observed in parietal and temporal regions than expected (χ^2^ statistic = 12.3, *p *=* *0.012).

HFA electrode groups also differed in their selectivity for specific movements (one-way ANOVA comparing mean activity during left hand vs right hand vs mouth; Fig. 3*E*). Movement-related electrodes most frequently showed distinct neural responses in relation to specific movements (47.1%, *n* = 16/34), followed by instruction-related electrodes (22.6%, *n* = 12/53), and HFA null electrodes (14.3%, *n* = 21/147; all more frequent than expected based on 5% false-positive rate, χ^2^ statistic = 22.2, *p < *0.001). We rarely observed instruction-related selectivity (χ^2^ test, *p > *0.5)

### Theta oscillations are widespread and independent of HFA

**Figure 4.**
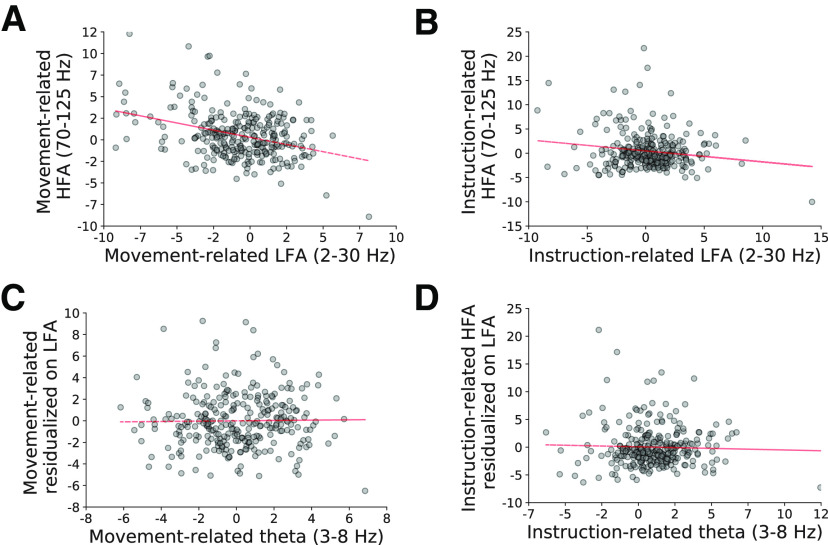
Task-related HFA is negatively related to wideband low-frequency activity (LFA, 2- to 30-Hz power), but independent of theta power. ***A***, ***B***, Scatter plots of cue-related HFA changes and LFA during the instruction (***A***) and movement (***B***) intervals. ***C***, ***D***, Scatter plots comparing HFA (residualized on LFA) and theta power changes during the instruction interval (***C***) and the movement interval (***D***). Dashed red line indicates line of best fit. See main text for statistics.

We found that cue-related HFA changes at each electrode were independent of narrowband theta power (3–8 Hz) after accounting of wideband frequency components of the power spectrum (“low-frequency activity,” 2- to 30-Hz power) using multivariate linear regression (Fig. 4). At each electrode, we measured instruction-related and movement-related measures of low-frequency activity and theta power using the same time intervals used to measure cue-related HFA (Materials and Methods). We found that movement-related changes in HFA were negatively related to low-frequency activity (*t* statistic of β coefficient = −3.68, FDR-corrected *p *=* *0.002), but independent of theta (*p *> 0.5). Instruction-related HFA changes were independent of both low-frequency activity and theta (*p*s > 0.25).

**Figure 5.**
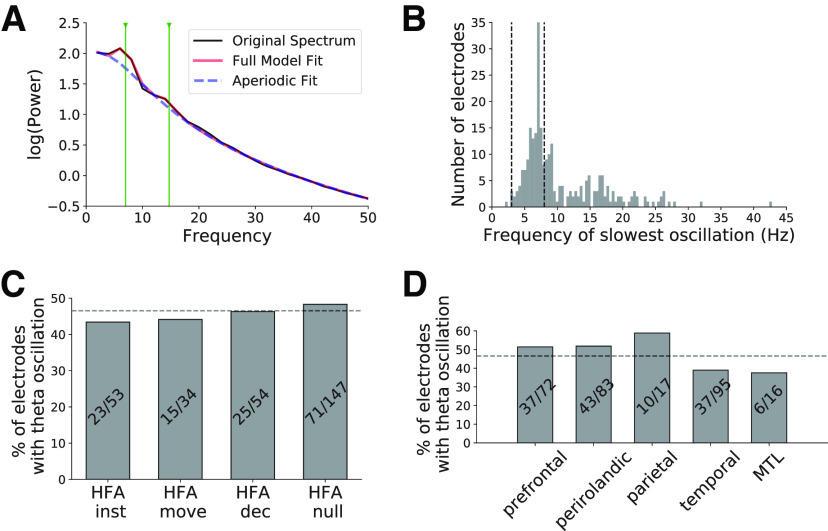
Theta oscillations are widespread throughout the brain. ***A***, Example power spectrum from a single electrode illustrating our method to identify oscillations. We applied a curve-fitting algorithm to model each power spectra as a combination of 1/*f* background (dashed blue line) and oscillatory peaks (black line shows original spectrum, red line shows model fit; see main text for details). Vertical green lines indicate the center frequency of detected spectral peaks (7 and 14 Hz) in this case. ***B***, Distribution of lowest frequency spectral peaks across all electrodes. We identified electrodes recording θ oscillations as those that showed a spectral peak with a center frequency between 3 and 8 Hz. Electrodes without a spectral peak are not shown. ***C***, ***D***, We show the distribution of these electrodes across HFA electrode groups (***C***) and various brain regions (***D***).

In a separate analysis, we identified electrodes that showed theta oscillations using a curve fitting procedure to detect 3- to 8-Hz peaks in the power spectrum beyond the background 1/*f* component (wideband low-frequency changes in the analysis above; Donoghue et al., 2020). We observed good fits to the power spectra across task-responsive electrodes (*R*^2^ = 0.99 ± 0.012; mean ± SD; Fig. 5*A*) and observed theta oscillations in 134/288 task-responsive electrodes (Fig. 5*B*). These oscillations were widespread across HFA electrode groups and brain regions (χ^2^ statistic, *p*s > 0.5; Fig. 5*C*,*D*). We focus the remainder of the analyses studying interactions among these electrodes that recorded theta oscillations.

### Pairwise theta synchrony is widespread but dependent on movement-related HFA

**Figure 6.**
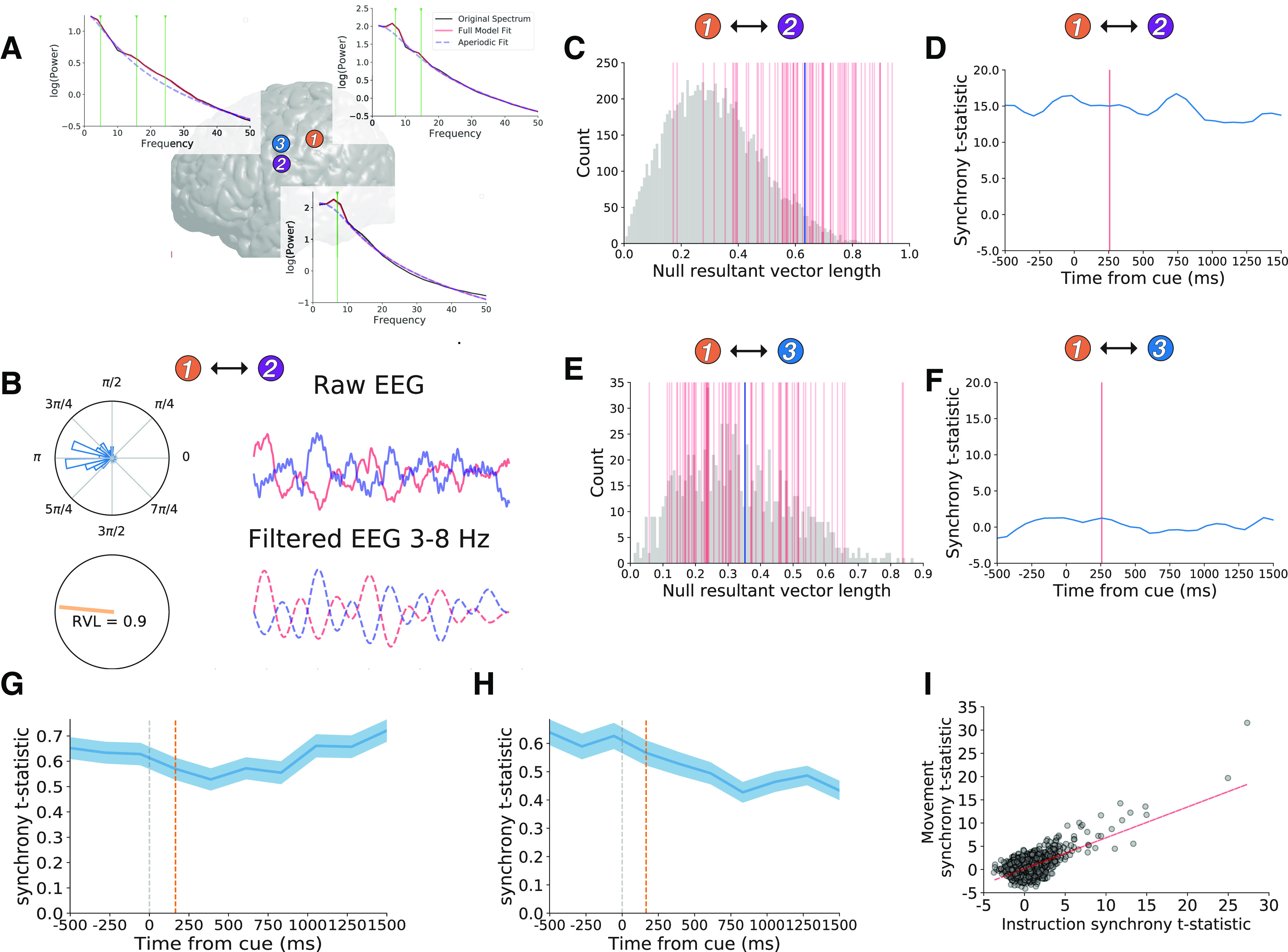
Theta oscillations are more synchronous than expected by chance. ***A***, We illustrate our method for measuring theta phase synchrony using three example electrodes from the perirolandic cortex (color scheme matched to Fig. 2). Brain plot showing locations of each electrode and power spectra with green lines marking theta oscillation center frequencies (6.99 Hz in electrode 1, 7.07 Hz in electrode 2, and 4.84 Hz in electrode 3). ***B***, Single trial data raw iEEG data (top, solid lines) and filtered 3- to 8-Hz iEEG (bottom, dashed lines) from electrode 1 (blue) and electrode 2 (red) during the instruction interval of interest (250 ms before and 750 ms after cue presentation). Polar plots show distribution of theta phase differences during this time interval showing a clustering around π. Orange line shows RVL (drawn on unit circle), which measures the non-uniformity or (tightness) of the phase distribution (RVL = 0.9 for this time interval). ***C***, Null distribution of RVL values generated from random resampling of contiguous 1-s phase data from electrodes 1 and 2. Vertical red lines mark true RVL values from each trial. Blue vertical line marks the mean true RVL distribution. We quantified the extent to which phase synchrony across trials differed from the null distribution via unpaired *t* test resulting in a synchrony *t* statistic for the time interval. ***D***, We show synchrony *t* statistics for the interaction between electrodes 1 and 2 surrounding several 1-s time intervals surrounding the instruction cue (−1000 ms before 2000 ms after using a sliding window analysis). Horizontal axis tick labels indicate the mean of each time window. Red line marks the mean of the instruction interval of interest (−250 to 750 ms). ***E***, ***F***, Same as ***C***, ***D*** for interactions between electrode 1 and electrode 3. We observed significant synchrony between electrode 1 and electrode 2 (synchrony *t* statistic = 14.9, *p *<* *0.001), but not between electrodes 1 and 3 during the instruction interval of interest (synchrony *t* statistic = 1.4, *p *>* *0.15). ***G***, ***H***, Mean of synchrony *t* statistics over time across all electrode pairs (*n* = 1807) during the instruction (***G***) and movement intervals (***H***), with red lines marking the instruction and movement intervals of interest. We observed greater synchrony across electrode pairs than expected by chance (see main text for statistics). Width indicates SEM across electrode pairs. ***I***, We observed positive correlation between instruction synchrony *t* statistics and movement-related synchrony *t* statistics across all electrode pairs.

We observed widespread pairwise phase synchrony between task-responsive electrodes that showed theta oscillations. For each pair of electrodes, we measured the extent to which theta oscillations were “coupled” over time by testing whether theta phase differences were more consistent than expected by chance. We focused on two time intervals that we hypothesized as potentially behaviorally relevant: 250 ms before, and 750 ms following, the instruction and movement cues. In each time interval, we quantified the extent to which phase coupling was greater than expected by chance, resulting in an instruction synchrony *t* statistic and movement synchrony *t* statistic. We illustrate our method for quantifying pairwise theta synchrony using three example electrodes from perirolandic cortex that were all task-responsive and showed theta oscillations (Fig. 6). We only included task-responsive electrodes with simultaneous iEEG recordings that both showed theta oscillations (*n* = 1806 electrode pairs)

We found that average pairwise theta synchrony was greater than expected by chance during both the instruction and movement intervals (paired *t* tests on distributions of average synchrony *t* statistics for each subject; instruction: *t*_(8)_ = 4.42, *p *=* *0.002; movement: *t*_(8)_ = 2.5, *p *=* *0.037). We observed significant (*p *<* *0.05) pairwise instruction-interval synchrony in 213/1806 electrode pairs and movement-interval synchrony in 253/1806 electrode pairs (χ^2^ tests, *p*s < 0.001; *n* = 90 expected by chance during each interval). We also found a positive correlation between theta synchrony during instruction and movement (Pearson *r* across pairs, *r = *0.65, *p *<* *0.001; and across subjects, *r *=* *0.71*, p *=* *0.029), suggesting that theta synchrony was generally stable throughout each trial and not driven by event-related phenomena.

**Figure 7.**
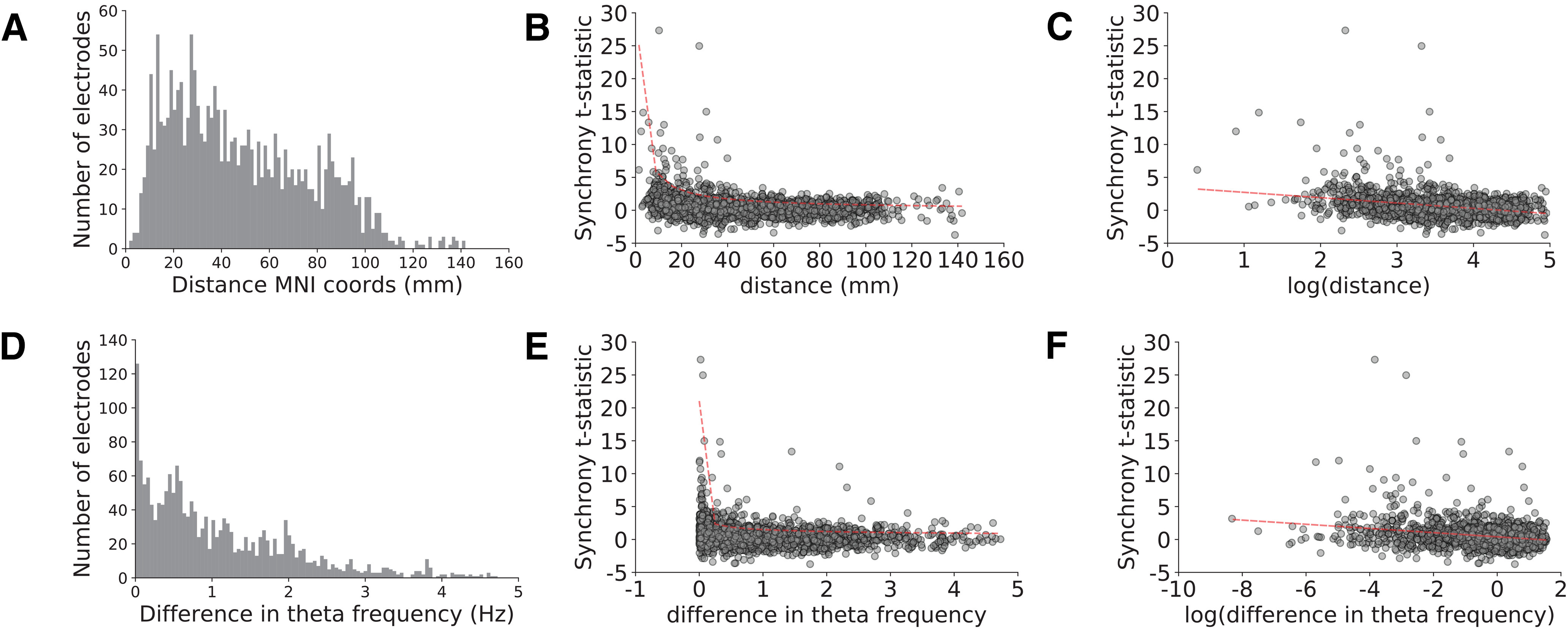
Theta synchrony is heavily influenced by physical distance and similarity in oscillation frequency. ***A***, ***D***, Distributions of Euclidian distance in MNI coordinate space (mm, ***A***) and differences between center frequency of theta oscillations (***D***) across electrode pairs. We show the relation between synchrony during the instruction interval and these measures but observed similar relations during the movement interval (see main text). ***B***, ***E***, Synchrony showed supra linear increases as a function of decreasing physical distance (***B***) and decreasing differences in theta center frequencies (***E***) that were fit by power law functions. ***C***, ***F***, Synchrony showed linear relations to log transforms of physical distance (***C***) and differences in theta frequency (***F***). Dashed red line shows line of best fit in each case.

We found that theta synchrony was heavily influenced by the physical distance between electrodes in a pair and the similarity in the frequency of theta oscillations detected at each electrode. In both cases, we observed a supra-linear increase in pairwise synchrony when electrodes were closer to each other in physical space and theta frequency (Fig. 7). We observed linear relations between pairwise synchrony and log transforms of each of these measures (Pearson’s *r *>* *0.25, *p *<* *0.001).

We found that theta synchrony was positively related to movement-related HFA increases after accounting for the effects of physical distance and theta similarity using multivariate linear regression. We fit two separate linear models for the instruction and movement time intervals. During the instruction interval, we asked whether instruction synchrony *t* statistics were dependent on mean instruction-related HFA observed at the electrode pair, whereas during the movement interval, we studied the relation between movement synchrony *t* statistics and mean movement-related HFA. In both models, we included log-transformed physical distance and log-transformed frequency similarity as additional independent variables.

Theta synchrony showed an independent, significant positive relation to cue-related HFA changes during the movement interval (*t* statistic of β coefficient = 2.10, FDR-corrected *p *=* *0.041), but not during the instruction interval (*p *>* *0.15). During both time intervals, theta synchrony was heavily dependent on physical proximity (*t* statistic of β coefficients > 11.1, FDR-corrected *p*s < 0.001) and theta frequency similarity (*t* statistic of β coefficients > 7.3, FDR-corrected *p*s < 0.001). In a *post hoc* analysis, we found that instruction-related synchrony also showed a weak positive relation with movement-related HFA (*t* statistic of β coefficient = 1.97, uncorrected *p *=* *0.049).

**Figure 8.**
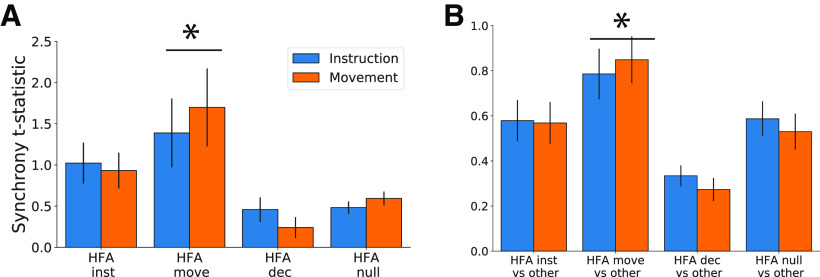
Theta synchrony was greater near movement-related electrodes. We show mean pairwise theta synchrony during the instruction (blue) and movement (orange) time intervals in relation to HFA electrode groups. ***A***, Average within-group synchrony for each group (interactions between electrode pairs where both electrodes were members of the group). ***B***, Average out-of-group synchrony for each group (interactions between electrode pairs where only one electrode was a members of the group). Note the different vertical axis range for ***A***, ***B***. Error bars indicate SEM across electrode pairs; * indicates *p *<* *0.05 for *post hoc* test comparing movement-related electrodes (HFA move) versus all other groups.

Consistent with these results, movement-related electrode group (HFA move) showed increased within-group theta synchrony (between two movement-related electrodes) and out-of-group theta synchrony (movement-related electrode vs non-movement-related electrode) relative to the remaining three HFA electrode groups during both the instruction (one-way ANOVA by group, *F* statistic > 4.9, *p*s < 0.003, *post hoc t* test movement-related electrodes vs all other groups *t*s > 2.5, *p*s < 0.012; Fig. 8) and movement intervals (one-way ANOVA by group, *F* statistic > 6.6, *p*s < 0.001, *post hoc t* test movement-related electrodes vs all other groups *t*s > 3.43, *p*s < 0.001, all *p*s FDR-corrected; Fig. 8).

## Discussion

We studied iEEG recordings obtained as patients performed a simple instructed movement task. Our main goal was to test the hypothesis that theta oscillations synchronize regions containing neural populations that are behaviorally relevant for voluntary movement. We used HFA (70- to 200-Hz power), a known proxy of local firing rates, to identify electrodes that were positioned near neural populations that showed temporally specific task-related neural activity (Manning et al., 2009; Ray and Maunsell, 2011; Dubey and Ray, 2019), and studied their anatomic relation to synchronous theta oscillations.

We found that HFA identified heterogenous neural response functions throughout the brain, including distinct responses from nearby brain regions. These results suggest that HFA is local neural signal akin to measuring multiunit activity from neural populations near the electrode (Dubey and Ray, 2019). We grouped electrodes based on their response functions to study distributed neural populations that shared activity patterns. We observed two prominent patterns of HFA increases. First, we identified instruction-related group that showed temporally specific increases in activity following the instruction cue, and second, a movement-related group that showed temporally specific increases in activity during movement. Instruction-related electrodes were widely distributed across regions involved in the ventral visual stream (occipital, temporal) and goal-directed and movement-planning networks (prefrontal and perirolandic regions), suggesting a relation to heterogeneous functions that occur during instruction presentation (sensory processing, goal-related and movement planning. On the other hand, movement-related electrodes were heavily clustered in perirolandic regions, suggest a prominent sampling of movement-generating neural processes We most frequently observed neural activity that distinguished between specific movements (left hand vs right hand vs mouth/tongue) at movement-related electrodes, consistent with a role in selecting specific movements, consistent with prior studies (Crone et al., 1998, 2006; Miller et al., 2007; Schalk et al., 2008; Cogan et al., 2014; Korzeniewska et al., 2015). We rarely observed neural activity that distinguished between specific instructions, which is consistent with recent findings in non-human primates suggesting that population-level neural representations of specific movements only emerge during movement generation (Kaufman et al., 2014; Elsayed et al., 2016).

A challenge in measuring theta oscillations is that narrowband theta power can conflate periodic theta oscillations and aperiodic low frequency power changes that are thought to reflect a distinct underlying neural process (Voytek and Knight, 2015; Donoghue et al., 2020). Consistent with this view, we found, via multivariate regression, that movement-related HFA changes were related to wideband low frequency power decreases, but independent of theta power. These results are consistent with a power spectrum “tilt” that has been widely observed in iEEG studies (Miller et al., 2007; Burke et al., 2015; Solomon et al., 2017), and may be related to “β” desynchronization that has been observed in scalp and intracranial EEG studies (Murthy and Fetz, 1992; Crone et al., 1998, 2006; Hari et al., 1998; Ohara, 2002; Jenkinson and Brown, 2011). However, instruction-related HFA changes were not related to wideband low frequency power, suggesting that HFA increases are not always accompanied by a power spectrum tilt. Further studies are needed to study the behavioral and structural underpinnings of spectral tilt in relation to HFA (Voytek and Knight, 2015; Gao et al., 2017; Herweg et al., 2020).

We observed theta oscillations at widespread electrodes throughout the brain, independent of local HFA changes following instruction or movement. We measured theta oscillations at each electrode by assessing whether the power spectrum contained narrowband peaks beyond the background 1/*f* shape using a recently described curve-fitting algorithm (Donoghue et al., 2020). This method allowed us to identify electrodes that showed periodic theta oscillations beyond asynchronous low frequency power changes. These results suggest that theta oscillations reflect a global neural signal, in contrast to HFA that measures local neural activity.

We found that these theta oscillations showed more phase synchrony over time than expected by chance. We measured phase synchrony over time in 1-s intervals throughout the trial rather than across trials in specific time windows to measure ongoing oscillations rather than event-related phenomena (Cohen and Donner, 2013; although see David et al., 2006). We found that theta synchrony was largely stable throughout each trial suggestive of ongoing oscillations, and in contrast to HFA that showed prominent within-trial dynamics.

We focused on phase-phase relations rather than other connectivity measures such as Granger causality or spectral coherence to mitigate the influence of asynchronous power correlations on our analyses (Lachaux et al., 1999; Herweg et al., 2020) and to relate our findings to previous studies of theta synchrony (Lega et al., 2012; Burke et al., 2013; Voytek et al., 2015; Solomon et al., 2017, 2019; Donoghue et al., 2020). In keeping with recent literature, we define “synchrony” between two signals to imply any periodically coupled temporal relationship, but not to imply a phase difference of zero, as would be expected perfectly coupled oscillators (Mirollo and Strogatz, 1990). Instead, our definition allows for variable offset phase differences as might be expected from multiple uncoupled oscillators or traveling waves (Zhang et al., 2018).

Theta synchrony showed a linear increase as a function of logarithmic decreases in physical distance which is consistent with previous findings (Lachaux et al., 1999) and may suggest a small-world structure to network interactions (Buzsáki, 2006; Bassett et al., 2018). Taken together with the global distribution of theta oscillations, and slow fluctuations across trials, these data suggest that theta synchrony may reflect dynamic functional connectivity between brain regions (Bickel et al., 2018), rather than event related neural activity. We also observed increased theta synchrony between electrodes that showed similar theta oscillation frequencies, which is consistent with the view that multiple oscillations are multiplexed within the theta frequency range (Jacobs, 2014).

After accounting for the effects described above, we found increased theta synchrony involving movement-related electrodes (that showed movement-related increases in HFA). This result supports the view that synchrony between theta oscillations plays a role in facilitating interactions between widespread behaviorally relevant neural populations during action selection (Cavanagh and Frank, 2014; Herweg et al., 2020). Our results provide an important complement to recent scalp EEG data showing a behavioral link between theta oscillations and sensory and motor functions (Tomassini et al., 2017). By showing that theta oscillations synchronize regions containing neural populations that are active when initiating instructed movement, our data provide an anatomic link between theta oscillations and movement-related neural populations in the human brain. Our data build on a large body of body of literature linking theta oscillations to human behavior in various domains, including perception (VanRullen, 2016), attention (Helfrich et al., 2018; VanRullen, 2018), spatial navigation (Jacobs, 2014), memory (Herweg et al., 2020), and decision-making (Cavanagh and Frank, 2014), and disease (Cavanagh et al., 2017; Singh et al., 2018).

In conclusion, we studied intracranial neural recordings patients with drug-refractory epilepsy performed a simple instructed motor task. We found that HFA measured distinct neural responses from nearby neural populations, suggesting a local signal. In contrast, theta oscillations were widespread and synchronous, suggesting a global neural signal. Theta synchrony was increased near neural populations that showed movement-related increases in local activity, suggesting that theta oscillations coordinate distributed neural representations during action selection.

### Future directions

Future studies should assess how theta synchrony fluctuates over time in relation to bottom-up arousal systems (Joshi et al., 2016; Stitt et al., 2018) and top-down cognitive control signals (Cavanagh et al., 2012) during higher cognitive functions such as learning (Montague et al., 1996; Ramayya et al., 2015), decision (Gold and Shadlen, 2007) and memory (Ratcliff, 1978), and assess mechanistic interactions to local neural populations via phase amplitude coupling (Galifianakis et al., 2013; Helfrich et al., 2018). Additionally, the application of graph-theoretic methods may be useful in studying global changes in theta synchrony in relation to cognitive states (Bassett et al., 2018).

### Limitations

We did not measure reaction times during the task and have limited ability to relate these neural signals to specific behavior. We had sparse electrode coverage across the nine subjects and have a limited ability to make claims about specific anatomic correlations of the observed neural responses beyond the region of interest analysis performed in the study. The opportunity to obtain human intracranial neurophysiology requires studying patient populations that may show systematic differences in neural structure and function relative to healthy individuals. In this case, increased functional connectivity from epilepsy (Bettus et al., 2008) may contribute to overestimating theta synchrony. However, it is unlikely to explain the HFA results, or the relation between theta synchrony and movement-related HFA.

## Synthesis

Reviewing Editor: Mark Laubach, American University Decisions are customarily a result of the Reviewing Editor and the peer reviewers coming together and discussing their recommendations until a consensus is reached. When revisions are invited, a fact-based synthesis statement explaining their decision and outlining what is needed to prepare a revision will be listed below. The following reviewer(s) agreed to reveal their identity: Nandakumar Narayanan, Charles Wilson.

Two reviewers and I have reviewed your paper submitted to eNeuro. The consensus was to request revisions with a second round of review. Details are provided in the following synthesis. Please be sure to address each point raised by each reviewer. Also, please consider using estimation statistics in your revised submission. More information about estimation statistics can be found here: https://doi.org/10.1523/ENEURO.0259-19.2019

---------------------------------------

Comments from Reviewer #1

eN-CFN-0252-20 analyzes widespread theta synchrony during instructed movement. The findings are important in our understanding of brain frequency dynamics. I had some questions that might help me understand the results and conclusions better:

1) Would readers benefit from an understand of the behavior - i.e., when the movements occurred relative to instruction? On line 530, it notes that RTs weren’t measured - is there any behavior? If not, I’d suggest pointing this out in the methods...

2) I didn’t understand how many electrodes went into data for 1c - ‘perirolandic’ can include many? Furthermore, I wonder if, as an exemplar, voltage domain data might be helpful in illustrating the analyses? What data went into the ‘right panel’?

3) For Figure 3 - would it help to visualize the ‘movement’ and ‘instruction’ group - essentially, visualizing Table 1 / Fig 3b?

4) I didn’t understand the phase-synchrony analysis. Why not use a more canonical approach - coherence or granger causality or some other metric? To be clear, I’m not asking for a new analysis, simply an explanation of this technique vs. other techniques, and why it was selected. Does this technique have a time-domain? It’d be interesting to see the theta peak in Fig 4b plotted in a time-frequency plot, but I didn’t know if this was possible. I think including a specific electrode pair in a specific subject with real data - particularly when matched to Figure 1 - might make it clear for me. Including these metrics alongside more traditional analyses might also help clarify things for me.

5) Would a table of subject data, and which contributed to which analyses help readers understand the dataset better?

6) There are diseases such as schizophrenia, anxiety, and Parkinson’s disease in which theta rhythms (or 4 Hz rhythms) are abnormal, and involve impaired sensory-motor integration. This includes work by the Cavanagh and Narayanan labs. Would this literature help the authors’ argument?

7) Was there any cingulate / medial prefrontal coverage?

8) Was there any difference between types of movements (r/l hand or tongue)?

Minor:

1) What is the italicized t on Figure 1a?

2) Is z-power the same as z-score (legend vs. axis label)

3) Line 35: Some would argue that there is a clear literature on theta and cognitive control as well as spatial memory

4) Line 96 - were seizure foci excluded?

5) Line 115 - was everything downsampled to 250 Hz? Does that affect Nyquist limits in Figure 1?

6) Is this data-driven PCA approach novel?

7) Line 284 - why was p<0.01 used here?

8) Line 361 - I wasn’t sure what figure this was related to...

---------------------------------------

Comments from Reviewer #2

The authors present human neurophysiological recordings in a very simple instructed movement task. They tag sites as showing modified high frequency activity at instruction and movement phases of this task, and then provide evidence for more consistent phase differences between instruction and movement sites just after movement initiation. The manuscript is well written and clear, but some more detail and a reconsideration of certain analyses is needed.

1. There is a lack of detail in the section describing the electrodes and their localisation. The section is entitled “electrocorticographic recordings” and yet there is mention of depth electrodes as well. The anatomical localisation section only discusses the surface electrodes. We have no detail on the nature of the electrodes themselves (size, material, etc.). A diversity of electrode type and localisation is to be completely expected in this sort of study, but that diversity should be described in detail, and its impact tested - especially if some electrodes are epicortical and some intracortical.

2. Why is the spectral power z-transformed by the mean/sd of random clips (line 147), rather than, say, the whole recording?

3. The procedure of “assigning” a frequency band label to each cluster (line 175) reposes strongly on the cited frequency ranges, but we know that these “bands” have significant individual differences. Wouldn’t it be better to provide the actual data, i.e. a central or median frequency of the cluster? (see also comment on theta band below).

4. I’m unclear what the PCA approach (line 188 onwards) is bringing here, compared to a simpler analysis that would nevertheless be data-driven to identify “movement” and “instruction” electrodes.

a) I think the justification for it could be better explained.

b) The PCA appears to be performed across all of the data from the subjects combined, yet the differences between subjects, for example electrode type, are not included in the analysis, and so their capacity to explain clusters remains unknown.

c) PCA group 2 shows significant power decreases in HFA for both instruction and movement, if I am reading Table 1 correctly, yet it is excluded from the analysis, as only HFA increases are sought. It’s unclear why the authors are only interested in HFA increases. It would be worthwhile understanding what this group is doing as well - they are clearly task related.

5. The procedure for testing theta synchrony (line 222 onwards) is restricted to a 400ms selected window, but a wide frequency definition of 3-8Hz is used. This means that there will be 1-3 cycles of theta, depending on the frequency. This brings up 2 important points:

a) The wide frequency band combined with low frequencies makes the phase synchrony difficult to interpret. Let’s imagine a case where we have a pair of electrodes with, say, 3Hz theta in one electrode and an 8Hz theta in another. So there would be nearly 3 cycles at 8Hz during the one 3Hz cycle. In this case it is hard to intuitively understand what a phase difference would be - during each 3Hz cycle you pass through each 8Hz phase 3 times. At higher frequencies this problem is much less apparent - between a 33Hz and a 38Hz oscillation, for example, and obviously if the frequencies are closely matched (e.g. 7Hz theta in both electrodes of a pair), their being in synchrony is intuitively understandable. The analysis here is built on this latter understanding, but does not account for the case of very discrepant frequencies. But I think the authors should consider how their analysis is treating such cases (I would guess it returns null phase differences when the frequencies are too far apart), and consider the impact of frequency matching between pairs on their analysis.

b) Restricting the analysis to a tight time window exacerbates this problem. Given that the analysis has been performed on a 3600ms time window, why not present all of the results, or better still a measure of time-resolved phase difference? For example the 100ms for processing time of the cue is fairly arbitrary, why not show us what is happening during this time? This might also help address the following point.

c) At such low frequencies in such short periods for analysis, it is difficult to determine whether the captured “theta” activity reflects some oscillation (the term employed throughout, presumably meaning an induced multicycle event) or merely an evoked deflection that simply approximates 1 low-frequency cycle. There are various treatments of this question in the literature, including the proposition to subtract evoked components before time-frequency analysis (e.g. Cohen, M. X., & Donner, T. H. (2013). Midfrontal conflict-related theta-band power reflects neural oscillations that predict behavior. Journal of Neurophysiology, 110(12), 2752-2763. http://doi.org/10.1152/jn.00479.2013). Alternative approaches argue that the evoked/induced distinction is not the best way to mechanistically think of the problem (e.g. David, O., Kilner, J. M., & Friston, K. J. (2006). Mechanisms of evoked and induced responses in MEG/EEG. NeuroImage, 31(4), 1580-1591. http://doi.org/10.1016/j.neuroimage.2006.02.034). Either way, I think the question should be addressed here. The point is, it is important to know exactly what the phenomenon that we are studying in the signal is.

6. I’m confused by the paragraph starting on line 343. The “double dissociation” is clear from Fig 3, but I don’t see why a 3-way interaction cited for a 3-fixed-effect ANOVA is support of this specific dissociation. The subsequent t statistics (with the opposing pair of tests added) would be more appropriate.

7. The pairwise phase difference analysis studies only pairs of electrodes with one instruction-related and one movement-related electrode. The text doesn’t make clear why this restriction is imposed, though I can imagine a couple of reasons. So first it should be made clear. Second, would not providing the same analysis for movement-movement and instruction-instruction pairs not provide an important contrast to contextualise the result presented? Without this the analysis feels unbalanced.

Minor points:

Line 232: “This our duration”?

Fig 2 legend refers to A) and D), rather than B)

## Author Response

We appreciate the reviewers’ insightful feedback and feel as though our manuscript is much improved as a result. We summarize the major changes to the manuscript and then provide a point-by-point response to each reviewer’s comments.

We group electrodes based on distinct instruction- and movement-related HFA changes, rather than using Principal Components Analysis and K-means clustering. We adopted a simpler approach as suggested by Reviewer 2 which has improved the interpretability of our results

We detect theta oscillations at each electrode using a recently described approach to model the power spectrum using parametric curve-fitting (Haller, et al, 2018). We used this approach to address Reviewer 2’s concerns about studying synchrony between theta oscillations with different center frequencies. We now limit our synchrony analysis to electrodes that show theta oscillations in the power spectrum and study the effect of similarity in theta frequency on synchrony measures.

We study theta synchrony over time in 1 second intervals, rather than across trials in narrow time intervals, in response to Reviewer 2’s concerns about measuring synchrony in narrow time windows and the potential confound of event-related phenomena.

We study pairwise interactions between all electrodes that show theta oscillations, and no longer limit our analyses to instruction and movement related electrodes, in response to Reviewer 2’s suggestion.

Comments from Reviewer #1

eN-CFN-0252-20 analyzes widespread theta synchrony during instructed movement. The findings are important in our understanding of brain frequency dynamics. I had some questions that might help me understand the results and conclusions better:

1) Would readers benefit from an understand of the behavior - i.e., when the movements occurred relative to instruction? On line 530, it notes that RTs weren’t measured - is there any behavior? If not, I’d suggest pointing this out in the methods...

We were not able to perform behavioral analyses as reaction times were not recorded during the task. We explicitly state this in the Methods section under “Instructed movement task"

2) I didn’t understand how many electrodes went into data for 1c - ‘perirolandic’ can include many? Furthermore, I wonder if, as an exemplar, voltage domain data might be helpful in illustrating the analyses? What data went into the ‘right panel’?

We have updated Figure 1 to improve clarity

We show data from an example electrode to illustrate our method of extracting HFA and theta from voltage time series and selecting task responsive electrodes.

3) For Figure 3 - would it help to visualize the ‘movement’ and ‘instruction’ group - essentially, visualizing Table 1 / Fig 3b?

We now illustrate the anatomical distribution of movement and instruction related electrodes in Figure 2

4) I didn’t understand the phase-synchrony analysis. Why not use a more canonical approach - coherence or granger causality or some other metric? To be clear, I’m not asking for a new analysis, simply an explanation of this technique vs. other techniques, and why it was selected. Does this technique have a time-domain? It’d be interesting to see the theta peak in Fig 4b plotted in a time-frequency plot, but I didn’t know if this was possible. I think including a specific electrode pair in a specific subject with real data - particularly when matched to Figure 1 - might make it clear for me. Including these metrics alongside more traditional analyses might also help clarify things for me.

We have clarified our methodology by expanding our description of the phase synchrony analysis in the methods section and illustrate the phase synchrony analysis in Figure 4 using example electrodes matched to Figure 2.

We have expanded the discussion section to better explain our rationale for using this technique.

Briefly, we wanted to use a pairwise connectivity measure that specifically measured theta phase coupling, as this neural process has been hypothesized as a neural mechanism of sensory-motor coordination (Cavanagh et al 2014, Herweg et al 2019). Coherence and Granger Causality are less specific measures of phase coupling because they can also be influenced by correlations in asynchronous power. We modeled our methods after a recent study that showed theta phase synchrony in the medial temporal lobe is important during memory retrieval (Solomon et al 2018). We wanted to study the importance of this neural process during sensory motor coordination.

5) Would a table of subject data, and which contributed to which analyses help readers understand the dataset better?

We now include subject data in Table 1 illustrating how each subject contributed to the analysis. We report group level properties of HFA electrodes in Figure 3 and 5, rather than in table form.

6) There are diseases such as schizophrenia, anxiety, and Parkinson’s disease in which theta rhythms (or 4 Hz rhythms) are abnormal, and involve impaired sensory-motor integration. This includes work by the Cavanagh and Narayanan labs. Would this literature help the authors’ argument?

We have included this point in the discussion section

7) Was there any cingulate / medial prefrontal coverage?

We did not have any electrodes that were localized to the cingulate or medial prefrontal cortex in this dataset

8) Was there any difference between types of movements (r/l hand or tongue)?

We describe how HFA differs between specific movements in the results section (Figure 3). We rarely observed differences in theta phase synchrony in relation to specific movements. However, because we do not have reaction time data, we are limited in our ability to study a temporal dependence of movement on theta phase.

Minor:

1) What is the italicized t on Figure 1a?

We report italicized p values in Figure 1 d that refers to non-parametric p-values associated with the two contiguous time frequency power changes shown (high frequency power increase and low frequency power decrease). We show the null distribution that was used to generate these p values.

2) Is z-power the same as z-score (legend vs. axis label)

Yes, we have updated the figures to improve consistency

3) Line 35: Some would argue that there is a clear literature on theta and cognitive control as well as spatial memory

We have revised this sentence

4) Line 96 - were seizure foci excluded?

We did not exclude seizure foci from this analysis. We have expanded the discussion section to discuss the inherent limitations of studying brain activity in patient populations and discuss our findings in the context of epileptiform brain activity (Limitations)

5) Line 115 - was everything downsampled to 250 Hz? Does that affect Nyquist limits in Figure 1?

We updated our definition of high frequency activity to be 70 - 125 Hz so that we are below the Nyquist frequency in all subjects.

6) Is this data-driven PCA approach novel?

We have replaced the PCA approach with a more direct measurement of evoked HFA activity as suggested by reviewer 2

7) Line 284 - why was p<0.01 used here?

This was an arbitrary statistical threshold value. We now use a p threshold of 0.05 to maintain consistency across the analyses.

8) Line 361 - I wasn’t sure what figure this was related to...

We have updated this section to described results from the revised analyses.

---------------------------------------

Comments from Reviewer #2

The authors present human neurophysiological recordings in a very simple instructed movement task. They tag sites as showing modified high frequency activity at instruction and movement phases of this task, and then provide evidence for more consistent phase differences between instruction and movement sites just after movement initiation. The manuscript is well written and clear, but some more detail and a reconsideration of certain analyses is needed.

1. There is a lack of detail in the section describing the electrodes and their localisation. The section is entitled “electrocorticographic recordings” and yet there is mention of depth electrodes as well. The anatomical localisation section only discusses the surface electrodes. We have no detail on the nature of the electrodes themselves (size, material, etc.). A diversity of electrode type and localisation is to be completely expected in this sort of study, but that diversity should be described in detail, and its impact tested - especially if some electrodes are epicortical and some intracortical.

We have updated the methods section to provide additional details about electrodes. We observed cue-evoked HFA changes and theta oscillations in regions that exclusively had intraparenchymal depth electrodes (MTL), exclusively had surface electrodes (parietal, perirolandic, occipital), suggesting that both electrode types contributed to our findings.

2. Why is the spectral power z-transformed by the mean/sd of random clips (line 147), rather than, say, the whole recording?

We followed methods used in prior intracranial EEG studies (Burke et al 2013, Ramayya et al 2015). We used random clips to reduce computational processing required to perform time frequency analyses on the entire recording. This method should not introduce a systemic bias in the resulting normalized values.

3. The procedure of “assigning” a frequency band label to each cluster (line 175) reposes strongly on the cited frequency ranges, but we know that these “bands” have significant individual differences. Wouldn’t it be better to provide the actual data, i.e. a central or median frequency of the cluster? (see also comment on theta band below).

In our revised analysis, we identified oscillations at each electrode by detecting peaks in the power spectrum (Haller et al 2018). As suggested, we report center frequencies of spectral changes observed across electrodes (Figure 5) prior to focusing on electrodes that showed theta oscillations (center frequency between 3 and 8 Hz).

We only use the cluster-based statistic procedure to identify task-responsive electrodes. We do not study center frequencies of low frequency clusters as these power changes are largely driven wideband low frequency changes, rather than theta oscillations (Figure 4)

4. I’m unclear what the PCA approach (line 188 onwards) is bringing here, compared to a simpler analysis that would nevertheless be data-driven to identify “movement” and “instruction” electrodes.

a) I think the justification for it could be better explained.

b) The PCA appears to be performed across all of the data from the subjects combined, yet the differences between subjects, for example electrode type, are not included in the analysis, and so their capacity to explain clusters remains unknown.

c) PCA group 2 shows significant power decreases in HFA for both instruction and movement, if I am reading Table 1 correctly, yet it is excluded from the analysis, as only HFA increases are sought. It’s unclear why the authors are only interested in HFA increases. It would be worthwhile understanding what this group is doing as well - they are clearly task related.

As suggested, we used a simpler analysis to identify movement and instruction related electrodes.

We also expanded our analyses to study task-responsive electrodes that showed HFA decreases or did not show task-related HFA changes.

• We have updated our analysis as follows. First, we first identified “task-responsive” electrodes that showed a power difference between task conditions at any frequency using a cluster-based permutation procedure. We then grouped these electrodes based on HFA changes observed following the instruction cue and the movement cue. We identified four groups of electrodes based on distinct patterns of tasked-evoked HFA activity: 1) HFA increases following instruction (instruction-related), 2) HFA increase following movement (movement-related), 3) HFA decrease following instruction or movement (HFA decrease) and 4) No evoked HFA change following instruction or movement (HFA null).

5. The procedure for testing theta synchrony (line 222 onwards) is restricted to a 400ms selected window, but a wide frequency definition of 3-8Hz is used. This means that there will be 1-3 cycles of theta, depending on the frequency. This brings up 2 important points:

a) The wide frequency band combined with low frequencies makes the phase synchrony difficult to interpret. Let’s imagine a case where we have a pair of electrodes with, say, 3Hz theta in one electrode and an 8Hz theta in another. So there would be nearly 3 cycles at 8Hz during the one 3Hz cycle. In this case it is hard to intuitively understand what a phase difference would be - during each 3Hz cycle you pass through each 8Hz phase 3 times. At higher frequencies this problem is much less apparent - between a 33Hz and a 38Hz oscillation, for example, and obviously if the frequencies are closely matched (e.g. 7Hz theta in both electrodes of a pair), their being in synchrony is intuitively understandable. The analysis here is built on this latter understanding, but does not account for the case of very discrepant frequencies. But I think the authors should consider how their analysis is treating such cases (I would guess it returns null phase differences when the frequencies are too far apart), and consider the impact of frequency matching between pairs on their analysis.

b) Restricting the analysis to a tight time window exacerbates this problem. Given that the analysis has been performed on a 3600ms time window, why not present all of the results, or better still a measure of time-resolved phase difference? For example the 100ms for processing time of the cue is fairly arbitrary, why not show us what is happening during this time? This might also help address the following point.

c) At such low frequencies in such short periods for analysis, it is difficult to determine whether the captured “theta” activity reflects some oscillation (the term employed throughout, presumably meaning an induced multicycle event) or merely an evoked deflection that simply approximates 1 low-frequency cycle. There are various treatments of this question in the literature, including the proposition to subtract evoked components before time-frequency analysis (e.g. Cohen, M. X., & Donner, T. H. (2013). Midfrontal conflict-related theta-band power reflects neural oscillations that predict behavior. Journal of Neurophysiology, 110(12), 2752-2763. http://doi.org/10.1152/jn.00479.2013). Alternative approaches argue that the evoked/induced distinction is not the best way to mechanistically think of the problem (e.g. David, O., Kilner, J. M., & Friston, K. J. (2006). Mechanisms of evoked and induced responses in MEG/EEG. NeuroImage, 31(4), 1580-1591. http://doi.org/10.1016/j.neuroimage.2006.02.034). Either way, I think the question should be addressed here. The point is, it is important to know exactly what the phenomenon that we are studying in the signal is.

The reviewer raises many important points concerning the phase synchrony analysis.

In response, we have made significant changes to our theta synchrony analysis

We only analyze electrodes that show theta oscillations in the power spectrum (Figure 5)

We measured across-time phase synchrony in 1 second clips (following methods from Solomon et al 2019), rather than across-trial phase synchrony in narrow time windows

We study the time course of pairwise phase synchrony in relation to task events using a sliding window analysis

We study how mismatches in the oscillatory frequency at each electrode influences pairwise synchrony measures.

Together, these changes improve the interpretability of the pairwise phase synchrony measurement and mitigate the influence of event-related potentials on the measurement

6. I’m confused by the paragraph starting on line 343. The “double dissociation” is clear from Fig 3, but I don’t see why a 3-way interaction cited for a 3-fixed-effect ANOVA is support of this specific dissociation. The subsequent t statistics (with the opposing pair of tests added) would be more appropriate.

We do not report this result in our revised analysis but acknowledge the reviewer’s point.

7. The pairwise phase difference analysis studies only pairs of electrodes with one instruction-related and one movement-related electrode. The text doesn’t make clear why this restriction is imposed, though I can imagine a couple of reasons. So first it should be made clear. Second, would not providing the same analysis for movement-movement and instruction-instruction pairs not provide an important contrast to contextualise the result presented? Without this the analysis feels unbalanced.

As suggested, we expanded our pairwise connectivity analysis to include interactions within and between each of the four electrode groups defined based on task-evoked HFA activity.

Minor points:

Line 232: “This our duration”?

Fig 2 legend refers to A) and D), rather than B)

We corrected these errors
